# COVID-19 Staff Wellbeing Survey: longitudinal survey of psychological well-being among health and social care staff in Northern Ireland during the COVID-19 pandemic

**DOI:** 10.1192/bjo.2021.988

**Published:** 2021-08-31

**Authors:** Julie-Ann Jordan, Ciaran Shannon, Dympna Browne, Emma Carroll, Jennifer Maguire, Keith Kerrigan, Sinead Hannan, Thomas McCarthy, Mark A. Tully, Ciaran Mulholland, Kevin F. W. Dyer

**Affiliations:** IMPACT Research Centre, Northern Health and Social Care Trust, Northern Ireland; Belfast Health and Social Care Trust, Northern Ireland; Institute of Mental Health Sciences, School of Health Sciences, Ulster University, Northern Ireland; Western Health and Social Care Trust, Northern Ireland; South Eastern Health and Social Care Trust, Northern Ireland; Southern Health and Social Care Trust, Northern Ireland

**Keywords:** Anxiety disorders, depressive disorders, post-traumatic stress disorder, sleep disorders, community mental health teams

## Abstract

**Background:**

Throughout the coronavirus disease 2019 (COVID-19) pandemic, health and social care workers have faced unprecedented professional demands, all of which are likely to have placed considerable strain on their psychological well-being.

**Aims:**

To measure the national prevalence of mental health symptoms within healthcare staff, and identify individual and organisational predictors of well-being.

**Method:**

The COVID-19 Staff Wellbeing Survey is a longitudinal online survey of psychological well-being among health and social care staff in Northern Ireland. The survey included four time points separated by 3-month intervals; time 1 (November 2020; *n* = 3834) and time 2 (February 2021; *n* = 2898) results are presented here. At time 2, 84% of respondents had received at least one dose of a COVID-19 vaccine. The survey included four validated psychological well-being questionnaires (depression, anxiety, post-traumatic stress and insomnia), as well as demographic and organisational measures.

**Results:**

At time 1 and 2, a high proportion of staff reported moderate-to-severe symptoms of depression (30–36%), anxiety (26–27%), post-traumatic stress (30–32%) and insomnia (27–28%); overall, significance tests and effect size data suggested psychological well-being was generally stable between November 2020 and February 2021 for health and social care staff. Multiple linear regression models indicated that perceptions of less effective communication within their organisation predicted greater levels of anxiety, depression, post-traumatic stress and insomnia.

**Conclusions:**

This study highlights the need to offer psychological support to all health and social care staff, and to communicate with staff regularly, frequently and clearly regarding COVID-19 to help protect staff psychological well-being.

## Background

The coronavirus disease 2019 (COVID-19) pandemic represents one of the most significant global threats to societal, physical and mental health in over a generation. Evidence from representative community studies indicate that the general population in the UK have experienced clinical levels of a range of psychological symptoms, including anxiety (22%), depression (22%) and post-traumatic stress (17%).^1^ Unsurprisingly, these figures are elevated for UK healthcare workers because of the considerable professional demands placed on them over a long-term period, with estimates of ‘caseness’ (i.e. moderate-to-severe symptoms) at 27% for depression, 23% for general anxiety and 30% for post-traumatic stress symptoms^2^ throughout the early stages of the pandemic. During the same time period, lower caseness estimates for depression (15%) and anxiety (12%), but higher rates of post-traumatic stress (35%) were reported among medical and nursing staff in China. Exposure to unique stressors and wider organisational strain, including ‘moral injury’ a source of psychological distress related to clinical pressures and decision-making that violates a staff member's moral or ethical code^3^ may partially account for these enhanced mental health difficulties in healthcare staff.^2^

Data from previous outbreaks and the current COVID-19 crisis suggest that both organisational and individual factors can mitigate the psychological impact of the pandemic on health workers. Mental health burden can be offset by workplace measures such as clear communication; supportive team networks; access to adequate personal protection equipment (PPE); provision of relevant training for job role; and access to appropriate psychological support.^4,5^ Healthcare staff in front-line positions involving direct contact with patients with COVID-19 are also at higher risk of psychopathology.^6^ Moreover, such organisational variables are likely to interact with personal factors such as age; professional experience; personal coping styles; family exposure to COVID; and pre-existing psychological difficulties that influence vulnerability to distress.^5,7^

Despite the rapidly evolving literature base on COVID-19-related mental health difficulties in healthcare staff, there remain a number of gaps in empirical understanding. Several prominent studies have focused on a restricted number of healthcare professions (for example medics and nurses)^8^ as opposed to representative samples of the entire healthcare workforce, including neglected subgroups such as domestic and support services. There is also a widely acknowledged need to move away from stand-alone cross-sectional studies and towards longitudinal methodologies examining the mechanism and course of mental health symptoms in staff over time.^2^ Moreover, risk factors and protective buffers within healthcare staff and their parent organisations need to be identified and tracked in order to ensure the development of timely, nuanced staff well-being support strategies.

## Aims

A key aim of the present exploratory study was to examine the impact of organisational, demographic and profession-specific factors on mental health. Online survey methodology was used to measure the national prevalence of mental health symptoms in health and social care staff as well as other relevant individual and organisational factors. It provides findings from the first two time points (3 months apart) of a larger longitudinal study examining changes in staff well-being during and after the second wave of the COVID-19 pandemic.

## Method

### Participants and design

The COVID-19 Staff Wellbeing Survey was open to all health and social care staff working in Northern Ireland. In Northern Ireland, both health and social care are provided by one organisation, in contrast to England where healthcare services are provided by the National Health Service and social care by local councils. The design incorporated both cross-sectional and longitudinal elements and spans four time points: time 1 (November 2020), time 2 (February 2021), time 3 (May 2021) and time 4 (August 2021). The time point spacing was designed to cover anticipated phases of the pandemic (such as COVID-19 wave peaks, pre- and post-vaccine), minimise survey fatigue effects and allow for service development in response to findings between time points.

Two time points have been completed thus far with data collection taking place during 9–22 November 2020 (time 1) and 8–28 February 2021 (time 2). Staff were recruited via a broad range of methods including broadcast emails to all staff; emails to staff who left an email address at time 1; posts on staff twitter and Facebook; laminated posters in staff areas; and screensaver messaging. At the time of data collection, approximately 78 000 staff^9^ were employed in health and social care roles in Northern Ireland, and were therefore eligible to take part. Of these staff, the cross-sectional sample sizes were 3834 at time 1 (response rate 4.9%) and 2898 at time 2 (response rate 3.7%). At time 1, a total of 5385 staff started to complete the survey with 71% of these completing it – further examination highlighted that respondents gradually dropped out throughout the survey and no specific question was particularly associated with drop-out. Staff were given the option of leaving their email address at each time point to enable their responses to be linked over time; a longitudinal data-set was created comprising the 632 staff who submitted their email address at times 1 and 2.

### Measures

The COVID-19 Staff Wellbeing Survey collected a broad range of data including demographics; caring responsibilities; job satisfaction; psychological well-being; redeployment experiences; COVID-19 risk factors and exposure; environmental needs; communication; accessed mental healthcare services; and future psychological needs. The focus of this article is on the four psychological well-being outcome measures.

The constructs measured included anxiety (Generalised Anxiety Disorder-7; GAD-7),^10^ depression (Patient Health Questionnaire-9; PHQ-9),^11^ post-traumatic stress (Impact of Event Scale-Revised; IES-R)^12^ and insomnia (Insomnia Severity Index; ISI).^13^ Established cut-off scores were used to designate symptoms as moderate-severe on these measures: ≥10 for GAD-7 and PHQ-9; ≥26 for IES-R; and ≥15 for ISI.^6,10,11,13,14^ The participants were instructed to complete the IES-R with ‘respect to the COVID-19 outbreak’.

The following variables were used as predictor variables of psychological well-being in the regression analyses: occupation, gender, age, COVID-19 exposure; if they managed patients with COVID-19; if they have one or more risk factors for COVID-19 (such as diabetes); perceived effectiveness of communication by their organisation on COVID-19-related matters, if they were asked to consider a redeployment opportunity; and if vaccinated (time 2 only). All binary predictors were coded as follows: 0, no; 1, yes. Further details on the psychological well-being outcome and predictor variables are included in Supplementary Table 1 available at https://doi.org/10.1192/bjo.2021.988.

### Procedure

Respondents voluntarily completed the survey online via the Survey Mechanics platform. At time 2 they were instructed that they could take part even if they had not participated at time 1. The authors assert that all procedures contributing to this work comply with the ethical standards of the relevant national and institutional committees on human experimentation and with the Helsinki Declaration of 1975, as revised in 2008. All procedures involving human patients were approved by West of Scotland Research Ethics Service (REC reference 20/WS/0122). Participants indicated their consent to participate by clicking to start the questionnaire after reading the online information sheet. Participants were free to withdraw from the study at any stage while completing the questionnaire up until they clicked ‘submit’ at the end of the questionnaire. Both the information sheet and the final page of the questionnaire provided details of individuals they could contact regarding psychological well-being support.

### Statistical analysis

All analyses were conducted using IBM SPSS Statistics version 26 for Windows. The demographic profiles of the time 1 and 2 cross-sectional samples were compared using the χ^2^-test for categorical variables and independent *t*-tests for continuous variables. We used *t*-tests on the cross-sectional (independent *t*-tests) and longitudinal (paired *t*-tests) samples to examine change over time on total scores of the psychological well-being measures. Chi-square (cross-sectional samples) and McNemar (longitudinal sample) tests provided an assessment of change over time in the proportion of health and social care staff reporting moderate-to-severe depression, anxiety, post-traumatic stress and insomnia symptoms.

The purpose of the longitudinal analysis was to check if changes in the cross-sectional sample were replicable or likely because of differences in the composition of the samples at the two time points. Multiple linear regression models with simultaneous entry were then used to examine predictors of psychological well-being for the time 1 and 2 cross-sectional samples.

All survey questions were mandatory (except email address); hence there were no missing values on any of the variables reported here, except for age where a small number of impossible values were recorded (time 1, 0.3%; time, 2 0.3%). In the regression models that included age as a covariate, listwise deletion was used.

## Results

### Participant characteristics

Participant characteristics at time 1 (*n* = 3834) and 2 (*n* = 2898) for the cross-sectional samples are presented in Table 1. Statistical analyses indicated that the profiles of the cross-sectional samples were comparable for the two time points. The average age of both samples was 44 years, and at both time points the vast majority of respondents were female (82–83%). This pattern is in keeping with the health and social care staff census data^15^ that shows that women comprise four-fifths (79%) of the workforce. The age profile of the time 1 sample and the health and social care staff profile is reasonably similar (34 years and under, time 1, 23%, population data 28%; 35–44 years, time 1 28%, population data 26%; 45–54 years, time 1 33%, population data 27%; 55 years plus, time 1 16%, population data 19%. The samples at both time points were highly educated, with three-quarters (74–75%) reporting being educated to level four (such as university degree) or above.

**Table 1:** Participant characteristics of the time 1 and 2 participants

Variables	Time 1	Time 2	*P*
Age, years: mean (s.d.)	43.61 (10.5)	43.87 (10.7)	0.320
Gender, *n* (%)			
Male	664 (17.3)	486 (16.8)	0.372
Female	3161 (82.4)	2409 (83.1)	–
Non-binary	9 (0.2)	3 (0.1)	–
Occupation, *n* (%)			
Admin and clerical	1059 (27.6)	813 (28.1)	0.141
Ambulance	76 (2.0)	79 (2.7)	–
Care home	93 (2.4)	57 (2.0)	–
Estates	24 (0.6)	19 (0.7)	–
Medical	243 (6.3)	175 (6.0)	–
Dental	24 (0.6)	18 (0.6)	–
Nursing and midwifery	903 (23.6)	695 (24.0)	–
Professional and technical	771 (20.1)	618 (21.3)	–
Senior executive	27 (0.7)	27 (0.9)	–
Social services	548 (14.3)	348 (12.0)	–
Support services/user experience	66 (1.7)	49 (1.7)	–
Highest qualification, *n* (%)			
None	12 (0.3)	6 (0.2)	0.866
Level one	90 (2.3)	65 (2.2)	–
Level two	275 (7.2)	191 (6.6)	–
Apprenticeships	5 (0.1)	2 (0.1)	–
Level three	347 (9.1)	258 (8.9)	–
Level four or above	2836 (74.0)	2176 (75.1)	–
Other	269 (7.0)	200 (6.9)	–

At time 2, 84% of respondents reported that they had received at least one dose of a COVID-19 vaccine. Generally speaking, the longitudinal sample had a similar demographic profile to the cross-sectional sample (see Supplementary Table 2). However, with the longitudinal sample the over-representation of administrative and clerical, and professional and technical staff was greater, as was the underrepresentation of nursing and midwifery staff.

A large proportion of the participants worked in administrative and clerical (28%), nursing and midwifery (24%), and professional and technical (20–21%) roles. Compared with occupational distribution data for health and social care staff^15^ (figures exclude care home and senior executives) the achieved sample has good representation from most sectors. Groups of staff with more desk-based roles such as administrative and clerical (time 1 28% *v*. population data 19%) and professional and technical (time 1 20% *v*. population data 15%) who would have had greater access to computers, unsurprisingly tended to be over-represented in the survey whereas those with greater patient contact (such as nursing and midwifery (time 1 24% *v*. population data 33%) were underrepresented.

Support services/user experience were the most underrepresented in the present sample; this sector typically comprises approximately 10% of the health and social care workforce, five times the proportion achieved in time 1 of the COVID-19 Wellbeing Survey.

### Psychological well-being of staff at time 1 and 2

**Fig. 1:**
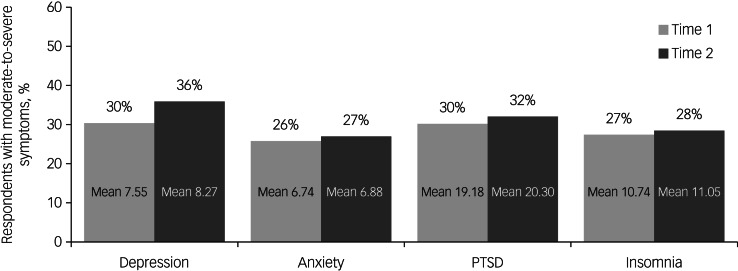
Proportion of respondents with moderate-to-severe symptoms in the time 1 and 2 cross-sectional samples. Mean scores are given for depression using the Patient Health Questionnaire-9; anxiety using the Generalised Anxiety Disorder-7; for post-traumatic stress disorder (PTSD) using the Impact of Event Scale-Revised; and for insomnia using the Insomnia Severity Index.

A high proportion of staff reported moderate-to-severe symptoms of depression, anxiety, PTSD, and insomnia in the time 1 (26–30%) and 2 (27–36%) cross-sectional samples (Fig. 1).

Comparisons of the cross-sectional samples (Supplementary Tables 3 and 4) revealed a significantly higher proportion of respondents reporting moderate-to-severe depression at time 2 than time 1 (χ2 = 22.51, d.f. = 1, *P* < 0.001, *d* = 0.12); no significant difference was evident for anxiety (χ^2^ = 1.15, d.f. = 1, *P* = 0.284, *d* = 0.03), post-traumatic stress (χ^2^ = 2.65, d.f. = 1, *P* = 0.104, *d* = 0.04) or insomnia (χ^2^ = 1.01, d.f. = 1, *P* = 0.315, *d* = 0.02).

The time 1 and 2 samples were also compared using the total scores on the four psychological well-being measures; significantly poorer well-being was evident in the time 2 sample compared with the time 1 sample for depression (*t*(6123.28) = −4.84, *P* < 0.001, *d* = 0.12), post-traumatic stress (*t*(6127.42) = −2.42, *P* = 0.016, *d* = 0.06) and insomnia (*t*(6730) = −2.06, *P* = 0.039, *d* = 0.05), but not for anxiety (*t*(6730) = −1.01, *P* = 0.312, *d* = 0.02). All comparisons between the cross-sectional samples yielded small effect sizes.

Comparable analyses were performed using the longitudinal sample (see Supplementary Tables 3 and 4). In keeping with the cross-sectional results, descriptive data for the longitudinal samples follow the trend of poorer well-being at time 2 compared with time 1. However, statistical analyses using McNemar tests showed no significant difference over time in the proportion reporting moderate-to-severe symptoms for depression (*P* = 0.071), anxiety (*P* = 0.694), post-traumatic stress (*P* = 0.863) or insomnia (*P* = 0.395) in the subsample for whom we had longitudinal data. Paired *t*-tests showed no significant change over time for depression (*t*(631) = −1.94, *P* < 0.053, *d* = 0.08), post-traumatic stress (*t*(631) = −0.45, *P* = 0.656, *d* = 0.02) or anxiety (*t*(631) = 0.01, *P* = 0.992, *d* = 0.00); a small but significant increase in insomnia symptoms was observed (*t*(631) = −2.34, *P* = 0.020, *d* = 0.09).

### Predictors of psychological well-being

Predictors of psychological well-being were considered at time 1 (Supplementary Table 5) and 2 (Table 2). Both sets of analyses considered identical predictors, except that the time 2 models also included if the participant had been vaccinated against COVID-19. All occupations were compared against those in nursing and midwifery roles, as this was one of the largest occupational groups in the sample and many of these staff would have been in front-line roles during the COVID-19 pandemic. The general pattern across the psychological well-being measures at times 1 and 2, indicated that nursing and midwifery staff have similar psychological well-being symptoms to ambulance, care home, estates, dental, senior executive and social services staff. Nursing and midwifery staff tended to have poorer psychological well-being compared with medical, and professional and technical staff, but better psychological well-being than support services staff. At time 1 only, administrative and clerical staff had greater anxiety, depression and post-traumatic stress than nursing and midwifery staff.

**Table 2:** Predictors of psychological well-being at time 2 (*n* = 2889)

	Depression		Anxiety		Post-traumatic stress disorder		Insomnia	
	*B* (s.e.)	*β*	*B* (s.e.)	*β*	*B* (s.e.)	*β*	*B* (s.e.)	*β*
Occupation								
Nursing and midwifery	Reference	–	Reference	–	Reference	–	Reference	
Administrative and clerical	0.01 (0.35)	0.00	0.47 (0.32)	0.04	1.45 (1.08)	0.03	0.30 (0.35)	0.02
Ambulance	−0.27 (0.72)	−0.01	0.54 (0.66)	0.02	1.23 (2.21)	0.01	−0.21 (0.72)	−0.01
Care home	−0.36 (0.81)	−0.01	−0.16 (0.75)	0.00	−2.38 (2.49)	−0.02	−0.12 (0.82)	0.00
Estates	−1.50 (1.39)	−0.02	−1.20 (1.28)	−0.02	−2.77 (4.26)	−0.01	−0.82 (1.40)	−0.01
Medical	−2.28 (0.51)	−0.09***	−1.66 (0.47)	−0.07***	−8.90 (1.55)	−0.11***	−3.32 (0.51)	−0.13***
Dental	1.40 (1.39)	0.02	1.77 (1.28)	0.02	3.98 (4.27)	0.02	0.88 (1.40)	0.01
Professional and technical	−1.53 (0.33)	−0.10***	−1.04 (0.31)	−0.07**	−3.97 (1.02)	−0.09***	−1.52 (0.34)	−0.10***
Senior executive	−0.99 (1.16)	−0.02	−0.61 (1.07)	−0.01	−7.24 (3.55)	−0.04*	−1.10 (1.16)	−0.02
Social services	−0.39 (0.40)	−0.02	−0.06 (0.37)	0.00	−0.43 (1.22)	−0.01	−0.25 (0.40)	−0.01
Support services/user experience	2.70 (0.88)	0.06**	2.33 (0.81)	0.05**	10.38 (2.69)	0.07***	3.06 (0.88)	0.06**
Gender								
Female	Reference	–	Reference	–	Reference	–	Reference	–
Male	0.08 (0.31)	0.00	−0.63 (0.29)	−0.04*	−1.28 (0.95)	−0.03	−0.28 (0.31)	−0.02
Non-binary	−1.71 (3.37)	−0.01	−3.84 (3.11)	−0.02	−11.87 (10.33)	−0.02	−7.48 (3.38)	−0.04*
Age, years	−0.10 (0.01)	−0.17***	−0.10 (0.01)	−0.18***	−0.14 (0.03)	−0.08***	−0.02 (0.01)	−0.03
Managed patients with COVID-19	0.24 (0.27)	0.02	0.36 (0.25)	0.03	2.01 (0.84)	0.05**	0.77 (0.27)	0.06**
Exposure to COVID-19	0.37 (0.08)	0.08***	0.35 (0.07)	0.09***	1.68 (0.24)	0.12***	0.40 (0.08)	0.09***
Have at least one COVID-19 risk factor	1.42 (0.26)	0.10***	0.96 (0.24)	0.07***	3.33 (0.80)	0.08***	1.63 (0.26)	0.11***
Perceived effectiveness of communication regarding COVID-19	−1.25 (0.11)	−0.22***	−1.13 (0.10)	−0.21***	−3.83 (0.32)	−0.22***	−1.11 (0.11)	−0.19***
If asked to consider being redeployed	0.54 (0.25)	0.04*	0.72 (0.23)	0.06**	2.36 (0.77)	0.06**	0.59 (.25)	0.04**
Received vaccine	0.39 (0.31)	0.02	0.00 (0.28)	0.00	−0.13 (0.94)	0.00	0.23 (0.31)	0.01

COVID-19, coronavirus disease 2019.

*P < 0.05, **P < 0.01, ***P < 0.001.

The *R*^2^ values for each of the models were: depression = 0.12***; anxiety = 0.12***; PTSD = 0.12***; insomnia = 0.10***

At both time points, a significant relationship was evident between at least two of the four psychological well-being measures and the organisational/risk factor variables. Specifically, poorer psychological well-being was associated with managing patients with COVID-19, having had higher exposure to COVID-19, having at least one COVID-19 risk factor, perceiving the communication from their organisation to have low effectiveness and being asked to consider a redeployment opportunity. Across both time and psychological well-being measures, the perceived effectiveness of communication by their organisation on COVID-19-related matters was the strongest predictor of well-being (*β* = −0.19 to −0.25).

## Discussion

### Main findings and comparison with findings from other studies

In this study, which was of a sample including all statutory health and social care organisations in a whole nation of the UK (Northern Ireland), we found high rates of depression, anxiety, post-traumatic stress and insomnia. It is the first study on healthcare staff to report longitudinal findings before and after staff have received their first vaccination. Across the two time points many staff reported moderate-to-severe levels of depression on the PHQ-9 (time 1, 30%, time 2, 36%), anxiety on the GAD-7 (time 1, 26%, time 2, 27%), PTSD on the IES-R (time 1, 30%, time 2, 32%) and of insomnia on the ISI (time 1, 27%, time 2, 28%). The results of cross-sectional analysis were broadly mirrored in the longitudinal analyses in that the psychological distress levels remained consistently high across the time points; where significant differences did occur the effect sizes were very small.

The rates reported here appear higher than those in the general UK and Irish populations during the first year of the pandemic.^1,16^ Shevlin et al^1^ report rates of moderate-to-severe depression on the PHQ-9 of 22%, that of anxiety on the GAD-7 of 22% and that of post-traumatic stress on the International Trauma Questionnaire of 17%. Our results are broadly in keeping with the higher end of estimates of caseness among healthcare workers elsewhere during the COVID-19 pandemic.

Worldwide studies have demonstrated very significant levels of anxiety, depression, insomnia and post-traumatic stress in healthcare workers with estimates of caseness ranging from 15 to 27% for depression, 12 to 23% for general anxiety and 30 to 35% for post-traumatic stress symptoms.^2,6^ However, we do note a recent review of populations affected by COVID^17^ that found no significant differences between healthcare workers and other populations affected by COVID-19 on measures of depression, anxiety and PTSD but twice the levels of insomnia – all groups in their analysis experienced much higher rates than would be expected.

In terms of predictors of distress, in keeping with previous literature,^18–20^ we found a range of individual and organisational variables have a role in predicting distress at both time points. Importantly, a strength of our sample was that it included all roles and jobs within the health and social care system in Northern Ireland. An important finding was that at time 1, administrative and clerical staff, and support services staff (such as cooks, cleaners, porters) had greater anxiety, depression and post-traumatic stress than nursing and midwifery staff. At both time points, we found a significant association between at least two of the four psychological well-being measures and the organisational/risk factor variables. Across both time and psychological well-being measures, the perceived effectiveness of communication by their organisation on COVID-19-related matters was the strongest predictor of well-being.

Vaccination uptake at time 2 did not predict well-being. It should be noted that the predictive models explained 10–12% of the variation in the four psychological well-being measures, meaning other factors not tapped by the models clearly contribute to staff well-being as well.

### Implications

The high rates of distress are in keeping with the need to provide interventions and prevention strategies to all types of healthcare workers both during this pandemic and as health systems are recovering from it. Despite the majority of our sample receiving their first vaccination at time 2 this did not appear to improve staff mental health. It appears organisations cannot rely on a vaccine ‘bounce’ to improve the well-being and mental health of their staff. While the evidence regarding effective staff support interventions is relatively sparse there is a need for intervention strategies to be developed at an individual, team and organisational level.^18,21^ Examples of interventions include psychological assistance hotlines, online courses and group activities to help with stress.^22^ Interventions may also include preventative approaches and the provision of timely and accessible individual mental health treatments in cases of emerging mental health problems.^23^

This study highlights that the provision of staff support interventions should not just be targeted at staff that are exposed to COVID-19 or that are working with patients with COVID-19. The results demonstrated that administration staff (secretaries and receptionists) as well as staff involved in support services (cooks, cleaners and porters) were at higher risk of distress than other staff groups. An effective health service needs a wide variety of jobs and roles to function effectively. It is imperative support interventions are available and accessible to all.

The findings are entirely consistent with a body of research highlighting the importance of organisational factors to staff well-being.^24,25^ This may very well be more important in a pandemic. By its very nature the situation is often entirely new to staff and guidance can change on a daily basis. Several professional bodies in the UK have highlighted the importance of a communication strategy to staff well-being and the importance of communicating with staff regularly, frequently and in simple clear ways.^26^ Muller et al,^21^ in a recent review, do note the frequent mismatch in studies of staff support interventions of the likely organisational sources of distress (communication, lack of PPE, workload) and the frequent focus on relieving distress at an individual level.

### Limitations

The starting point of this study was during the second wave of the pandemic (November 2020) and the second time point was February 2021. An obvious limitation is the lack of pre-pandemic baseline of staff mental health. However, as stated earlier we can compare rates with a number of studies of the general population in the UK and Ireland during the pandemic.^1,16^ Although there have been few psychiatric epidemiological studies in Northern Ireland to compare our rates with, the one exception is rates of PTSD. The Northern Ireland Study of Health and Stress, part of the World Mental Health Survey Initiative previously reported levels of PTSD in Northern Ireland of 5%.^27^ Our current rates of PTSD, as measured by the IES-R are considerably higher.

It is strength of our study that we included all staff groups. However, there was low uptake from some occupations (such as support services) meaning that the rates cannot be used as precise ‘prevalence rates’ for the whole of the health and social care sector. Rather they provide a general indication of the level of need. In staff surveys in Northern Ireland that were run pre-COVID-19, response rates have tended to be lowest in this sector, as they can be particularly hard to reach (i.e. no work email addresses). Engaging with this group during a pandemic has become even more challenging because of infection control rules (such as no postal option possible, strict rules on use of posters). Given that the group who were most underrepresented tended to have poorer mental health, the overall prevalence figure may be an underestimation of levels of distress among staff.

A further limitation is that our indicators of mental health are based on survey self-report data rather than diagnosis based on clinical interviews. We have, however, used instruments with good psychometric properties and our methodology is in keeping with all other studies of staff mental health during the pandemic that we are aware of. It should also be acknowledged that there is a lack of consensus regarding established clinical cut-offs for use with the IES-R; to allow for international comparisons we adopted that used by Lai et al.^6^

In conclusion, this study is one of the first longitudinal studies of health and social care staff mental health and well-being during the COVID-19 pandemic to be published. It strengthens the argument about the need to provide a comprehensive system of staff supports to all health and social care staff during and post this pandemic. This would appear essential if health services begin to recover function following this global pandemic.

## Data Availability

The data that support the findings of this study are available from the corresponding author (C.S.), upon reasonable request. Demographic data will be aggregated in order to protect the identity of the participants.

## Supplementary material

For supplementary material accompanying this paper visit http://dx.doi.org/10.1192/bjo.2021.988. click here to view supplementary material
