# Supplementary material for: COVID-19 Staff Wellbeing Survey: longitudinal survey of psychological well-being among health and social care staff in Northern Ireland during the COVID-19 pandemic
Source: BJPsych Open. 2021 Aug 31;7(5):e159. doi: 10.1192/bjo.2021.988 (PMC8410744; doi:10.1192/bjo.2021.988)
Supplement: Supplementary file 1 [file S2056472421009881sup001.docx]

***Supplementary materials***

Supplementary Table 1: Measures from the Staff COVID-19 Wellbeing Survey

| **Variable** | **Measurement details** | **Scale range** |
| --- | --- | --- |
| Depression | Measured by the Patient Health Questionnaire-9 (PHQ-9)^10^ This 9-item measure focuses on depressive symptoms in the last two weeks. The items use 4-point likert scales (‘not at all’ to ‘nearly every day’); higher scores are indicative of greater levels of depressive symptoms; minimal/no depression (0–4), mild (5–9); moderate (10–14); moderately severe (15–19); and severe (20-27) depression.  Present study Cronbach’s alphas: Time 1 (.90); Time 2 (.91) | 0-27 |
| Anxiety | Assessed by the Generalised Anxiety Disorder-7 (GAD-7),^9^ a 7-item measure of anxiety symptoms in the last 2 weeks. Items use 4-point likert scales (0=not at all, 3 = nearly every day), with higher scores indicating greater anxiety levels; minimal/no anxiety (0–4), mild anxiety (5–9), moderate anxiety (10–14), or severe anxiety (15–21).  Present study Cronbach’s alphas: Time 1 (.94); Time 2 (.94) | 0-21 |
| Post-traumatic stress | The 22 item Impact of Event Scale-Revised (IES-R)^11^ was used to ascertain levels of distress relating to the COVID-19 outbreak in the past seven days. The measure is DSM-IV compatible and uses 5-point likert items (0 = not at all; 4 = extremely) to measure distress in response to a specific life event, in this case COVID-19. Total scores are categorized as follows: subclinical (0-8), mild distress (9-25), moderate distress (26-43), and severe distress (44-88).  Present study Cronbach’s alphas: Time 1 (.96); Time 2 (.96) | 0-88 |
| Insomnia | Respondents insomnia symptoms over the last month were evaluated using the 7-item Insomnia Severity Index (ISI).^12^ All questions use 5-point likert scales with higher score indicative of great insomnia symptoms. The measure can be used classify insomnia severity to be classified as normal (0-7), subthreshold (8-14), moderate insomnia (15-21), or severe insomnia (22-28).  Present study Cronbach’s alphas: Time 1 (.91); Time 2 (90) | 0-28 |
| Occupation | Participants were asked to indicate which occupational category they belonged to.   1. *Admin & Clerical (e.g. clerical officer; HR; finance; ICT; health records; procurement, legal);* 2. *Ambulance;* 3. *Care home (e.g. care assistant; care home manager);* 4. *Estates (e.g. joiner, plumber, electrician, maintenance staff, estate officer);* 5. *Medical;* 6. *Dental;* 7. *Nursing & Midwifery;* 8. *Professional & Technical (e.g. staff working in pharmacy, laboratories, biomedical engineering; audiology; physiotherapy; dietetics; occupational therapy; speech and language therapy; radiography; podiatry; orthoptics, psychology other);* 9. *Senior executive;* 10. *Social Services (e.g. social worker, social care assistant, domiciliary care);* 11. *Support Services/User Experience (e.g. cooks and catering assistants; porters; domestic assistants; laundry workers; drivers)* | - |
| COVID-19 exposure | Sum of seven COVID-19 exposure-related questions:   1. Have you ever received a confirmed diagnosis of COVID-19? 2. Has anyone in your family ever received a confirmed diagnosis of COVID-19? 3. Have any of your friends ever received a confirmed diagnosis of COVID-19? 4. Have your neighbours (people living in the same community who may or may not know each other) ever received a confirmed diagnosis of COVID-19? 5. Has anyone who lives with you had a suspected (but unconfirmed) COVID-19 diagnosis? 6. Have you had a suspected (but unconfirmed) COVID-19 diagnosis? 7. Has someone that you know personally died as a result of COVID-19? | 0-7 |
| Managed patients with COVID-19 | Respondents were asked ‘have you ever managed patients with a confirmed diagnosis of COVID-19?’ | Yes=1  No=0 |
| Considered redeployment | At Time 1 the participants were asked ‘Have you been asked to consider taking on a redeployment opportunity due to the COVID-19 pandemic? At Time 2, the wording was adapted to refer to ‘in the last three months’ | Yes=1  No=0 |
| COVID-19 risk factor | HSC staff were asked to indicate which of the following risk factors for a severe course of COVID-19 applied to them: Older than 60 years; Cardiovascular disease; Diabetes; Immunodeficiency, or taking medication that suppresses the immune system (e.g. cortisone).; Chronic disease of the respiratory system (e.g. asthma, chronic bronchitis).; Chronic liver disease; Chronic kidney disease  Acute cancer; Cancer during past 5 years; Long-standing heavy cigarette consumption (more than 20 cigarettes per day in the last 5-10 years); None of the above risk factors. This question was taken from the COVID risk factor questions from the COVID-19 Pandemic Mental Health Questionnaire (CoPaQ)^28^  If they indicated yes for at least one risk factor this variable was coded (1, yes), otherwise it was coded as (0, No). | Yes=1  No=0 |
| Communication effectiveness | At Time 1, the COVID-19 Staff Wellbeing Survey included the following question ‘How effective has the communication about COVID-19 related matters been from your organisation?’. At Time 2, the wording was adapted to refer to ‘in the last three months. Responses were recorded on a 5 point likert scale ranging from not effective (0) to very effective (4). | 0-4 |
| Vaccine | Participants were asked ‘have you received one or both doses of a COVID-19 vaccine (e.g. Pfizer/BioNtech or Oxford/AstraZeneca).’ | Yes=1  No=0 |

Supplementary Table 2: Sample characteristics of the longitudinal sample at Times 1 and 2, data shown as n (%) unless otherwise stated

| Variables | Time 1 | Time 2 |
| --- | --- | --- |
|  |  |  |
| Age (mean, s.d.) | 43.44 (10.4) | 43.70 (10.4) |
| Gender |  |  |
| Male | 103 (16.3) | 102 (16.1) |
| Female | 527 (83.4) | 529 (83.7) |
| Non-binary | 2 (0.3) | 1 (0.2) |
| Occupation |  |  |
| Admin & Clerical | 204 (32.3) | 199 (31.5) |
| Ambulance | 7 (1.1) | 8 (1.3) |
| Care home | 9 (1.4) | 9 (1.4) |
| Estates | 1 (0.2) | 1 (0.2) |
| Medical | 40 (6.3) | 41 (6.5) |
| Dental | 2 (0.3) | 2 (0.3) |
| Nursing & midwifery | 111 (17.6) | 115 (18.2) |
| Professional & technical | 170 (26.9) | 170 (26.9) |
| Senior executive | 6 (0.9) | 5 (0.8) |
| Social services | 76 (12.0) | 76 (12.0) |
| Support services/user experience | 6 (0.9) | 6 (0.9) |
| Highest qualification |  |  |
| None | 1 (0.2) | 1 (0.2) |
| Level one | 10 (1.6) | 8 (1.3) |
| Level two | 41 (6.5) | 31 (4.9) |
| Apprenticeships | 0 (0.0) | 0 (0.0) |
| Level three | 47 (7.4) | 56 (8.9) |
| Level four or above | 490 (77.5) | 494 (78.2) |
| Other | 43 (6.8) | 42 (6.6) |

Supplementary Table 3: Mean total scores on the psychological wellbeing measures at Times 1 & 2

|  | Cross-sectional samples  (Time 1 n = 3,834; Time 2 n = 2,898) | | | Longitudinal sample (n = 632) | | |
| --- | --- | --- | --- | --- | --- | --- |
|  | Time 1  Mean  (SD) | Time 2  Mean  (SD) | *t(df), p* | Time 1 Mean (SD) | Time 2  Mean (SD) | *t(df), p* |
| Depression | 7.55  (5.97) | 8.27  (6.18) | t = -4.84(6123.28),  *p* < .001 | 7.23  (5.61) | 7.56  (5.67) | *t = -1.94 (631)*  *p* = .053 |
| Anxiety | 6.74  (5.76) | 6.88  (5.71) | t = -1.01 (6730),  *p* = .312 | 6.14  (5.34) | 6.14  (5.16) | *t = .01 (631)*  *p* =.992 |
| PTSD | 19.18  (18.31) | 20.30 (18.93) | t= -2.42 (6127.42),  *p* =.016 | 18.28  (16.65) | 18.53 (17.20) | *t = -.45 (631)*  *p* =.656 |
| Insomnia | 10.74  (6.24) | 11.05  (6.14) | t= -2.06 (6730),  *p* = .039 | 10.37  (5.85) | 10.76 (5.82) | *t = -2.34 (631)*  *p* = .020 |

Supplementary Table 4: Proportion with moderate to severe symptoms on psychological wellbeing measures at Times 1 & 2

|  | Cross-sectional samples  (Time 1 n = 3,834; Time 2 n = 2,898) | | | Longitudinal sample  (n = 632) | | |
| --- | --- | --- | --- | --- | --- | --- |
|  | Time 1 % | Time 2  % | X^2^ (df), *p* | Time 1 (%) | Time 2 (%) | *p* |
| Depression | 30.40 | 35.92 | X^2^ =22.51 (1), *p* < .001 | 28.64 | 32.12 | .071 |
| Anxiety | 25.80 | 26.98 | X^2^ =1.15 (1), *p =* .284 | 22.15 | 21.36 | .694 |
| PTSD | 30.20 | 32.06 | X^2^ = 2.65 (1) *p =* .104 | 28.64 | 29.11 | .863 |
| Insomnia | 27.40 | 28.47 | X^2^ = 1.01 (1) *p =* .315 | 24.53 | 26.11 | .395 |

Supplementary Table 5: Predictors of psychological wellbeing at Time 1 (n =3,822)

|  |  | Depression | | Anxiety | | PTSD | | Insomnia | |
| --- | --- | --- | --- | --- | --- | --- | --- | --- | --- |
|  |  | B(SE *B)* | *β* | *B (*SE *B)* | *β* | *B (*SE *B)* | *β* | *B (*SE *B)* | *β* |
| *Occupation* | *Nursing & Midwifery (reference)* |  |  |  |  |  |  |  |  |
|  | *Administrative & Clerical* | .61 (.29) | .05* | .79 (.28) | .06** | 2.47 (.88) | .06** | .57 (.31) | .04 |
|  | *Ambulance* | .80 (.69) | .02 | .20 (.66) | .01 | -1.22 (2.11) | -.01 | .67 (.73) | .02 |
|  | *Care home* | .33(.61) | .01 | .55 (.59) | .02 | -.27 (1.89) | -.00 | .86 (.65) | .02 |
|  | *Estates* | -.00 (1.19) | .00 | -.99 (1.14) | -.01 | -5.53 (3.65) | -.02 | .24 (1.26) | .00 |
|  | *Medical* | -1.97 (.42) | -.08*** | -1.63 (.40) | -.07*** | -7.45 (1.28) | -.10*** | -3.09 (.44) | -.12*** |
|  | *Dental* | -1.11 (1.16) | -.02 | -.74 (1.12) | -.01 | -6.53 (3.58) | -.03 | -1.37 (1.23) | -.02 |
|  | *Professional and technical* | -.79 (.29) | -.05** | -.63 (.28) | -.04* | -2.05 (.88) | -.05* | -1.43 (.30) | -.09*** |
|  | *Senior executive* | -1.43 (1.10) | -.02 | -.05 (1.06) | -.00 | -4.62 (3.40) | -.02 | -.06 (1.17) | -.00 |
|  | *Social services* | -.37 (.32) | -.02 | -.08 (.31) | -.01 | -1.33 (.98) | -.03 | .09 (.34) | .01 |
|  | *Support services/user experience* | 1.96 (.73) | .04** | 2.00 (.70) | .05** | 5.69 (2.24) | .04* | 1.73 (.77) | .04* |
| *Gender* | *Female (reference)* |  |  |  |  |  |  |  |  |
|  | *Male* | -.19 (.25) | -.01 | -.67 (.25) | -.04** | -1.92 (.78) | -.04* | -.50 (.27) | -.03 |
|  | *Non-binary* | 1.25 (1.87) | .01 | 1.23 (1.81) | .01 | 7.60 (5.76) | .02 | .63 (1.99) | .01 |
| *Age (years)* | | -.05 (.01) | -.10*** | -.06 (.01) | -.11*** | -.02 (.03) | -.01 | .01 (.01) | .01 |
| *Managed patients with COVID-19* | | .49 (.23) | .04* | .38 (.22) | .03 | 1.48 (.71) | .04* | .94 (.25) | .07*** |
| *Exposure to COVID-19* | | .47 (.07) | .11*** | .51 (.07) | .12*** | 1.87 (.21) | .14*** | .58 (.07) | .13*** |
| *Have at least one COVID-19 risk factor* | | 1.49 (.22) | .11*** | .84 (.21) | .06*** | 3.75 (.67) | .09*** | 1.29 (.23) | .09*** |
| *Perceived effectiveness of communication regarding COVID-19* | | -1.34 (.08) | -.25*** | -1.29 (.08) | -.25*** | -4.01 (.26) | -.25*** | -1.07 (.09) | -.19*** |
| *If asked to consider being redeployed* | | .80 (.20) | .06*** | .74 (.19) | .06*** | 1.42 (.61) | .04* | .36 (.21) | .03 |
| *R*^2^ | | .12*** | | .12*** | | .12*** | | .10*** | |

*p<.05 **p<.01 ***p<.001
